# Biomimetic Neurostimulation for the Enhancement of Lower-Limb Explosive Performance: A Pilot Study

**DOI:** 10.3390/jfmk11040398

**Published:** 2026-10-01

**Authors:** Igor Val Danilov, Agita Abele

**Affiliations:** 1 Scientific Institute, Riga Nordic University, LV-1019 Riga, Latvia; 2 Department of Sports Academy, Riga Stradins University, LV-1007 Riga, Latvia

**Keywords:** biomedicine, central fatigue, natural neurostimulation, neuromuscular fatigue, physical therapy, sports therapy

## Abstract

**Background**: Developing non-invasive bioengineering interventions to mitigate central fatigue remains a critical objective in applied physiology and sports medicine. This pilot study investigated a potential temporal association between a Natural Neurostimulation (NN) intervention and the short-term recovery of lower-limb explosive performance, utilizing a non-randomized, fixed-sequence design. **Method**: Athletes (*N* = 8) underwent pre- and post-exercise assessments under control (Day 1) and experimental NN (Day 2) conditions. Using a decoupled bilateral force-plate protocol, the study assessed lower-limb explosive performance recovery following upper-body loading. **Results**: Descriptive statistics demonstrated a mean Jump Height effect of 0.052 ± 0.034, with a 95% confidence interval (CI) [0.023, 0.081], and a mean Peak Power effect of 0.032 ± 0.035, with a 95% CI [0.002, 0.062]. For Jump Height, a paired-samples *t*-test comparing Day 1 and Day 2 retention coefficients revealed a statistically significant improvement (*t* = 4.284, *p* = 0.0036), indicating a very large intervention effect (Cohen’s *d_z_* = 1.515). Peak Power retention demonstrated a similarly significant improvement (*t* = 2.545, *p* = 0.0384), yielding a large effect size (Cohen’s *d_z_* = 0.900). **Conclusions**: The NN intervention was associated with statistically significant, large-magnitude improvements in both Jump Height and Peak Power retention coefficients following fatiguing exercise. While these findings suggest that the NN protocol may facilitate the short-term recovery of lower-limb explosive performance, the absence of randomization and a sham control precludes establishing a definitive causal relationship. Nevertheless, these preliminary results demonstrate the operational feasibility of the intervention, providing a clear methodological foundation for future fully powered, randomized controlled trials.

## 1. Introduction

Scientific literature indicates that physical fatigue in athletes engaged in repeated exercise is characterized by complex neuromuscular and metabolic dysfunctions, leading to a diminished capacity for muscular force production. This decline is classified by sports science into two interrelated mechanisms: peripheral fatigue, which manifests within muscle tissue, and central fatigue, which originates in the central nervous system [1,2,3]. Central fatigue has been extensively documented as a limiting factor in team sports performance. Accordingly, the development and identification of interventions to mitigate central fatigue are warranted [4,5]. The implementation of non-invasive engineering approaches that facilitate the attenuation of central fatigue would represent a significant advancement for applications in both biological and medical sciences.

The primary objective of this pilot study is to explore a potential temporal association between a Natural Neurostimulation (NN) intervention and the short-term recovery of lower-limb explosive performance following an upper-body loading protocol. To evaluate this, lower-limb explosive performance was assessed via dual force-plate mechanics to track central fatigue preservation following upper-body loading [6,7,8,9,10].

Natural neurostimulation (NN) refers to maternal neurostimulation of the fetal nervous system during pregnancy. The hypothesis proposes that maternal–fetal interactions promote balanced neurodevelopment and biological sentience in offspring. The heart is a powerful source of electromagnetic (EM) fields and acoustic waves in the body. Retain the theoretical concept that maternal heart tones (*S*_1_, *S*_2_, *S*_3_) and EM oscillations provide biomimetic templates, while removing the exact decibel and frequency values [11,12,13]. According to the NN hypothesis, maternal cardiac activity may facilitate temporal synchronization of neuronal populations in anatomically distinct maternal and fetal nervous systems. Acoustic and EM stimulation modalities interact with deep cranial structures, particularly during periods of task-induced brain activity, such as when the mother encounters social and environmental auditory stimuli that also reach the fetus. These EM fields and acoustic waves during cognitive load synchronize both nervous systems and stimulate neuronal activity in the fetus, forming early cortical architecture, maturing sensory pathways, and integrating large-scale neural networks [14].

Mimicking endogenous neurostimulation, as observed in maternal–fetal interactions, may represent a viable approach for mitigating central fatigue originating within the nervous system. The NN technique is postulated to enhance neural communication between the central nervous system and peripheral musculature [14]. Specifically, this NN procedure may restore homeostasis within the clinical immuno-neuro-endocrine axis by targeting neural regulation. Therefore, it influences the rhythmic release of inhibitory neurotransmitters and hormones [15]. Hormonal surges directly enhance signaling across the neuromuscular pathway connecting the central nervous system to peripheral skeletal muscles [15].

The potential mechanism underlying NN impact on the nervous system is assumed to involve optimizing mitochondrial bioenergetics. Mitochondria are highly proteome-efficient in generating adenosine-5′-triphosphate (ATP). ATP stores and transports energy in organisms [16,17]. Mitochondria are biophysical sensing hubs that continuously adapt their bioenergetic output (ATP generation, calcium buffering, and ROS signaling) in response to external forces [18]. Driven by physical mechanisms of non-convective energy transfer—such as mechanical work, electrical potentials, electromagnetic radiation, and thermal gradients—mitochondrial cellular respiration consumes oxygen to produce ATP, which fuels cellular functions and maintains synaptic neurotransmission [19]. Mitochondria, commonly found in synaptic terminals, play a crucial role in maintaining neurotransmission by producing ATP and buffering Ca^2+^. These cells contribute to a unique physicochemical set of actions that affect neuronal activity [19]. Therefore, the NN intervention is hypothesized to stimulate mitochondrial activity to restore homeostasis across the clinical immuno-neuro-endocrine (CINE) axis by bolstering neural regulation and downstream signaling pathways. Additionally, experimental evidence indicates that cardiac-mimicking acoustic and EM fields may improve cerebral oxygenation via vasodilation and stimulate adenosine triphosphate (ATP) release, ultimately promoting increased synaptic protein synthesis [14]. Collectively, the NN intervention is proposed to enhance neural connectivity and functional integration between central and peripheral components of the nervous system for mitigating central fatigue.

In this context, because the biomimetic approach involves systematic abstraction, modeling, and technological translation of evolutionarily conserved biological mechanisms, the Natural Neurostimulation (NN) paradigm abstracts key features of maternal–fetal neurostimulation to establish an optimized, synthetic technique. Rather than merely replicating endogenous maternal inputs, the NN technique adapts and optimizes this mechanism to function effectively within the scaled neurophysiological substrate of the mature organism. The NN system uses weak, dual-modality acoustic-electromagnetic stimulation, administered concurrently with task-induced brain activity [14]. The Section 2 and Section 4 present more details on the transition from the NN phenomenon to the NN intervention used in the experiments. They show the physiological rationale connecting maternal/fetal neurostimulation, visual and acoustic stimulation, mitochondrial activity, cerebral oxygenation, and reduced exercise-induced central fatigue.

## 2. Method

This pilot study investigated a potential temporal association between the NN intervention and the short-term recovery of lower-limb explosive performance. During the initial control condition (Day 1), lower-limb explosive performance—an established proxy for neuromuscular fatigue dynamics [6,7,8,9,10]—was assessed before and after a standardized upper-body loading protocol without neurostimulation. Following a 120-h washout period, the identical assessment sequence was repeated for the experimental condition (Day 2), with the NN intervention administered during the acute post-exercise recovery window.

The study used a VALD ForceDecks system (Brisbane, Australia) that evaluates neuromuscular fatigue by revealing deficits in how the brain communicates with muscles to produce maximum power [6,7,8,9,10]. When an individual is fatigued, their gross output may remain unchanged, but their movement strategy will shift dramatically to compensate [6,7,8,9,10]. Empirical evidence demonstrates that countermovement jump (CMJ) assessments on bilateral force-plate systems yield valid evaluations of neuromuscular fatigue [6,7,8,9] and enable the discrete identification of central fatigue metrics within a decoupled methodological framework [10] (see Section 4). Because the study aimed to measure the effect of NN on jumping performance associated with central fatigue, we applied a decoupled VALD methodology based on the phenomenon of non-local muscle fatigue (NLMF) [10]: we assessed athletes’ lower-limb kinematic metrics while loading their upper limbs. Thus, this decoupled design was selected to capture indirect indicators of central fatigue [10], as the lower limbs remained inactive during the upper-body physical load (see Section 4).

### 2.1. Participants

Ten male football players from a single squad of the RFS Academy (Riga, Latvia) took part in the study. On the first testing day, their age was 17.9 ± 1.2 years (range 16.8–20.4), and their body mass was 80.5 ± 7.1 kg (73.0–96.6). All were registered with the Latvian Football Federation and competed at an amateur level in national youth and reserve-team competition. Registered competitive football experience, defined as the time from each player’s first federation registration to the first testing day, was 9.6 ± 1.8 years (7.4–12.6); players had first been registered at 8.3 ± 1.0 years of age (6.7–9.7). All participants trained in the same squad under the same weekly program and the same coaching staff. Six of the ten participants were under 18 years of age at the time of testing.

Inclusion criteria were squad membership; medical clearance and freedom from injury or illness at the time of testing; and availability for both testing days. Participants were recruited by the head coach, who nominated athletes able to commit to both sessions; participation was voluntary and could be withdrawn at any time. Medical staff, including a physiotherapist, were present throughout both sessions, and we measured blood pressure before and after each session. Continuous monitoring ensured that multimodal stimulation (e.g., photonic stimulation, magnetic field emission, and active cognitive processing) did not cause clinical cardiovascular distress or unwanted side effects during testing.

Prior to enrollment in the study, all participants and their legal guardians received a comprehensive consent-form document detailing the study’s purpose, psychological demands, risks, benefits, data confidentiality, and voluntary nature. Athletes and their parents (in the case of minor athletes) signed written consent forms.

We recruited ten athletes, and they completed Day 1. Two of them did not attend Day 2 for reasons unrelated to the study, so paired Day 1 and Day 2 data were available for eight participants. Investigators excluded no participants, and no data were discarded.

The experiments were conducted five days apart, exactly 120 h. We selected these two days from the athletes’ schedules that had identical training schedules three days earlier. Both sessions started at the same time of day; the first jump trial was recorded between 08:34 and 08:42 on both days, and each athlete was tested at the same point in the session on both days. Participants wore the same clothing and footwear on both days and were instructed to arrive well rested, normally hydrated and fed, and to avoid additional strenuous training in the preceding 24–48 h. We carefully incorporated the experiments into athletes’ training schedules, balancing their load. Therefore, we standardized previous training, recovery duration, sleep, food intake, hydration, time of day, and strenuous exercise performed during the preceding 24–48 h. The team coach selected the physical load program and 1RM for the experiment based on the athletes’ anthropometric characteristics. The athletes were familiar with the VALD ForceDecks system; they used the VALD measurements regularly, using the standard VALD procedure.

### 2.2. Study Design and Procedure

The study applied a within-subject research paradigm: we observed indirect indicators of neuromuscular fatigue in each subject under two conditions: without/with neurostimulation. We administered experimental conditions in a single-arm, fixed-sequence procedure: a non-randomized, fixed-sequence design without a sham control arm. The Section 4 explains the methodological justification for using this design for the pilot study.

On the first day, we assessed lower-limb explosive performance after a 35-min physical load. To determine the physical load corresponding to 70–85% of the One-Repetition Maximum (1RM), maximum strength was initially quantified via direct 1RM testing. The target intensity boundaries were subsequently established by multiplying the baseline 1RM by the designated load coefficients (e.g., 0.70 × RM, corresponding to approximately 10–12 repetitions per set). Total physical load was calculated using the standardized volume load equation: Volume Load (kg) = Load (kg) × Repetitions × Sets.

Data collected on Day 1 established the control baseline for assessment of lower-limb explosive performance. Following a 120-h washout period, participants executed an identical exercise protocol on Day 2, with the sole experimental manipulation being the administration of the Natural Neurostimulation (NN) intervention post-exercise to facilitate neural recovery. Performance metrics from the experimental condition (Day 2) were subsequently compared against the control condition (Day 1) to evaluate the potential influence of the NN intervention on the short-term recovery of lower-limb explosive performance following upper-body loading.

#### 2.2.1. Day 1

Pre-Exercise Assessment: After a blood pressure check, athletes performed initial jump tests on the VALD ForceDecks platform to record baseline force-time curves. Exercise Session: The load training protocol (Table 1) lasted 35 min. Participants completed a series of intense physical exercises designed to induce standard athletic fatigue. We asked athletes to perform five intense weight-bearing exercises for the upper extremities. The load was set at 70% to 85% of the participant’s One-Repetition Maximum (One-RM).

The exact sequence of the steps in the Session (35 min) of 8 athletes:

▪ First Jump test T1 on the VALD ForceDecks platform (1 min);
▪ Exercise Round (35 min);
▪ Second Jump test T2 on the VALD ForceDecks platform (1 min);
▪ Pause 15 min. During the pause, participants rested in chairs;
▪ Third Jump test T3 on the VALD ForceDecks platform (1 min).

#### 2.2.2. Day 2

Pre-Exercise Assessment: After blood pressure check, athletes repeated the same baseline jump tests on the VALD ForceDecks platform. Exercise Session: Participants performed the exact same sports exercises (Exercise Round, see Table 1) as on Day 1. Natural Neurostimulation Procedure: Participants received a 15-min non-invasive NN session during the 15 min break after the round.

The exact sequence of the steps in the Session (55 min) for 8 athletes:

▪ First Jump test T1 on the VALD ForceDecks platform (1 min);
▪ Exercise Round (30–35 min);
▪ Second Jump test T2 on the VALD ForceDecks platform (1 min);
▪ Pause 15 min. During the NN procedure, participants sat in chairs;
▪ Third Jump test T3 on the VALD ForceDecks platform (1 min).

### 2.3. NN Intervention and Measurement Procedures

The NN technique delivers non-invasive neurostimulation via ubiquitous electronic hardware (e.g., smartphones) that generates extremely low-frequency electromagnetic fields (ELF-EMFs) with magnetic flux densities in the microtesla range (it is a million times larger than brain activity fields). At operational distances of 0.2–0.4 m, these ambient fields safely interface with central neural substrates, targeting cortical networks associated with conscious awareness through tuned sensory inputs. The photonic component of the NN paradigm is engineered to functionalize light delivery as an analog to cardiac electromagnetic field oscillations. Administered at a temporal repetition rate of 1.3 Hz, photic stimulation employs alternating spectral pulses between short-wavelength and long-wavelength light. This visual driving protocol is hypothesized to: induce cortical phase-locking via rhythmic photonic entrainment, maximize cortical power engagement across distinct visual processing pathways through alternating spectral extremes, and attenuate alpha-band inhibition while promoting localized gamma-band synchronization. Concurrently, cardiovascular-mimetic acoustic waveforms generate periodic acoustic oscillations that alter plasma membrane tension, thereby promoting the gating of mechanosensitive ion channels at the cellular interface.

Therefore, at the macro-level, administering the biomimetic neurostimulation concurrently with task-induced cognitive processing is hypothesized to drive temporal neuronal synchronization and recruit functional cognitive networks. At the cellular and micro-level, the NN intervention is proposed to modulate mitochondrial bioenergetics by optimizing non-convective energy transfer, adenosine triphosphate (ATP) synthesis, intracellular calcium (Ca^2+^) buffering, and reactive oxygen species (ROS) signaling—processes critical for maintaining sustained synaptic transmission. 

Furthermore, the NN technique is theorized to enhance cerebral oxygenation via endothelium-dependent vasodilation, upregulate synaptic protein synthesis, and modulate the rhythmic release of inhibitory neurotransmitters and endocrine signals. In the context of physical performance, these systemic neuroendocrine adjustments are proposed to directly enhance efferent neural drive along the central-to-peripheral neuromuscular axis. Consequently, these integrated neural pathways provide a plausible theoretical framework for the acute preservation of lower-limb kinetic output, offering a prospective mechanism for attenuating exercise-induced central fatigue. The Section 4 provides a mechanistic foundation for the NN technique rationale, drawing upon the broader literature.

#### 2.3.1. NN Intervention Procedure and Aparatus

**Figure 1:**
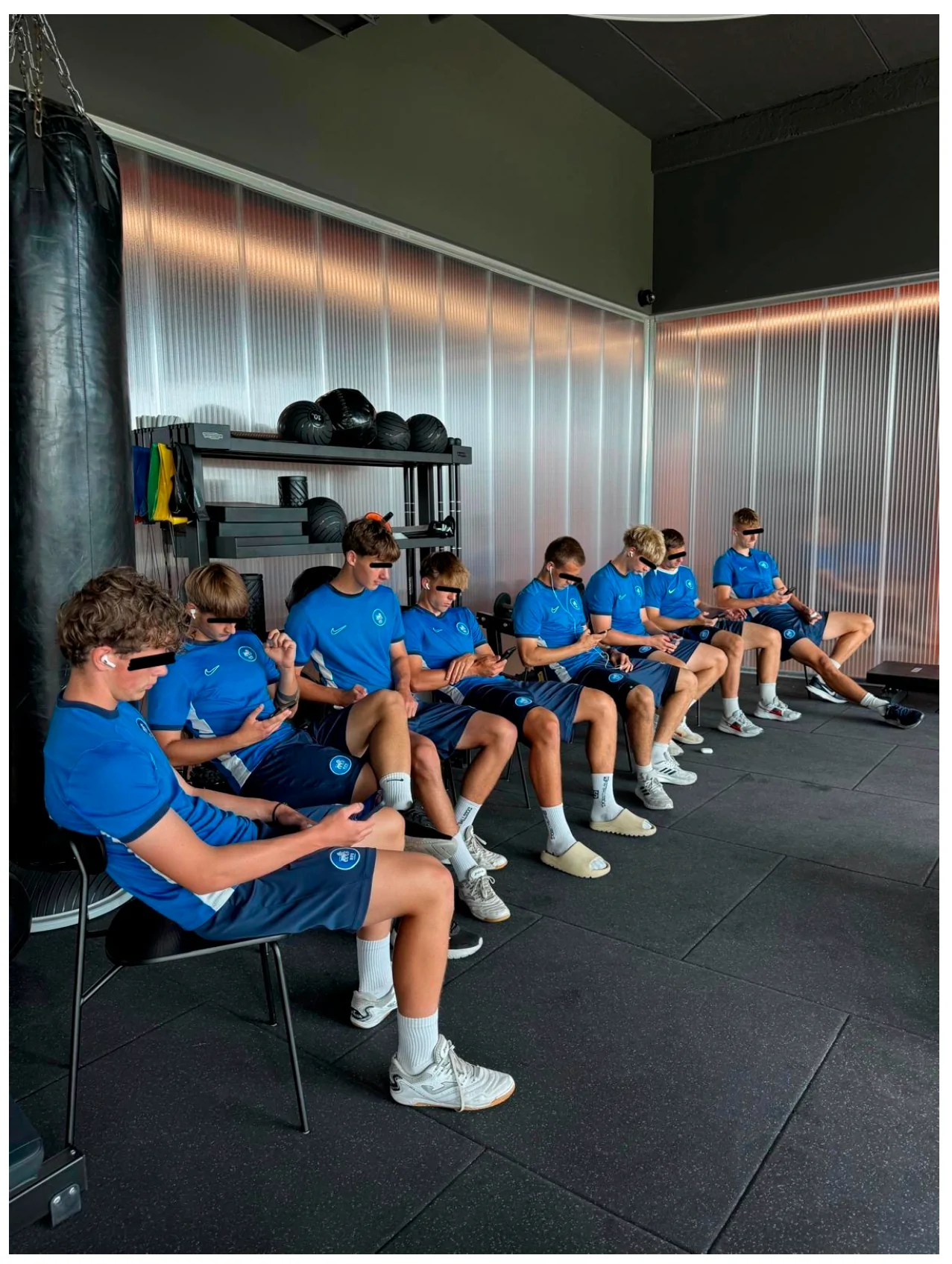
The subject receiving NN intervention.

During the Natural Neurostimulation (NN) intervention, participants were seated in an upright position holding a smartphone at a viewing distance of 0.2–0.4 m while wearing headphones (Figure 1). Upon accessing a web-based platform hosting the proprietary stimulation software, the multi-modal intervention was initiated. The stimulation protocol delivered concurrent visual, electromagnetic, acoustic, and cognitive stimuli specified as follows. Photic Stimulation: Visual driving was elicited via 450 lux alternating monochromatic pulses (violet at 400 nm and red at 700 nm) and operating at 1.3 Hz to drive visual brain stimulation. EM Emissions: 50–60 Hz frequency with a magnetic flux density of 0.13–0.3 μT and 0.08 μT magnetic flux density near the left and right temporal lobes. Acoustic Transduction: Specialized headphones emitting complex waves: frequency of 1.3 Hz operational frequency matching heart sinusoids and wave composition of three simultaneous sine waves (*S*_1_ at 70 dB; *S*_2_ and *S*_3_ at 21 dB). Cognitive Load: Active mental processing of 40 math problems.

#### 2.3.2. Measurement Procedures

The ForceDecks jump protocol of countermovement jump (CMJ) was performed on a VALD ForceDecks dual force plate system, without additional load, sampled at 500 Hz. Before the experiments, we checked that all athletes were familiar with this system procedure. We also instructed them on the main requirements before measurement: to step onto the center of the plates and stand completely still for 1 to 3 s (to calculate their body weight prior to movement). For the jump execution, we reminded them to stand in a normal, upright posture with hands placed firmly on the hips. The hands must remain anchored to the hips throughout the entire movement to eliminate upper-body momentum and isolate lower-limb kinetic outputs. Then, the athlete must keep their chest up and maintain a forward gaze. For the descent phase, the athlete should rapidly drop downward (bending the knees and hips) to a self-selected depth. For the takeoff without pausing at the bottom of the dip, the athlete should immediately and explosively drive upward, jumping as high vertically as possible. During the flight phase, the athlete must keep their knees fully extended (straight legs) while in the air. To land, the athlete must land softly on the force plates and immediately return to the quiet starting position. 

Owing to the participants’ prior familiarity with the bilateral force-plate protocol, a single trial per outcome measure was performed. Additional trial iterations raised our concerns about overloading the athletes with irrelevant exercises for the study design. Since the participants demonstrated precise technical execution and strict protocol adherence during the single-trial counterforce jump (CMJ) assessment, we excluded repetitions. Recent research has also shown that the single-trial countermovement jump protocol yields valid and highly dependable estimates for primary concentric output metrics, specifically jump height [20].

Jump assessments were conducted at baseline (T1), immediately post-exercise (T2), and after a 15-min recovery period (T3) each experimental day. However, the T2 results were excluded from the final analysis because immediate post-exercise measurements are confounded by acute physiological responses—namely, post-activation potentiation (PAP or PAPE) and surges in catecholamines—that temporarily mask neuromuscular fatigue [21,22,23]. In contrast, T3 (15 min post-exercise) serves as a critical time point, capturing recovery dynamics after these confounders have dissipated. Consequently, analysis of the T3/T1 retention ratio revealed distinct performance recovery dynamics when comparing the initial control day to the subsequent Natural Neurostimulation (NN) trial (see Section 4).

### 2.4. Outcome Measures

The retention coefficients were calculated using the following calculation logic: First, we calculated how short-term recovery of lower-limb explosive performance was altered owing to NN condition (the intervention during the 15-min recovery window on the 2nd day) by calculating a difference between the JH coefficient P(*N*)_1_^2^/P(*N*)_1_^1^ ratio on in the first day and the JH coefficient P(*N*)_2_^2^/P(*N*)_2_^1^ ratio on the second day for each athlete.

JH Coef*_N2_* = P(*N*)_2_^2^/P(*N*)_2_^1^ (1)

*N* is a number of participants, P(*N*)_2_^2^—second measurement in the second day, P(*N*)_2_^1^—baseline measurement in the second day,

JH Coef*_N1_* = P(*N*)_1_^2^/P(*N*)_1_^1^ (2)

and on the first day, P(*N*)_1_^2^—second measurement and P(*N*)_1_^1^—baseline measurement.

Second, we calculated how short-term recovery of lower-limb explosive performance was altered owing to the NN condition (the intervention during the 15-min recovery window on the 2nd day), by calculating the difference between the PP coefficient P(*N*)_1_^2^/P(*N*)_1_^1^ ratio on the first day and the PP coefficient P(*N*)_2_^2^/P(*N*)_2_^1^ ratio on the second day for each athlete.

PP Coef*_N2_* = P(*N*)_2_^2^/P(*N*)_2_^1^ (3)

PP Coef*_N1_* = P(*N*)_1_^2^/P(*N*)_1_^1^ (4)

A positive value (Equations (5) and (6)) indicates that, in the NN condition (Day 2), athletes improved their ability to retain baseline performance compared to the control day.

JH Effect(*N*) = JH Coef*_N2_* − JH Coef*_N1_* (5)

PP Effect(*N*) = PP Coef*_N2_* − PP Coef*_N1_* (6)

### 2.5. Statistical Analysis

All statistical analyses were conducted using Python (v3.11.5, SciPy statistical library v1.11.2). The primary objective of the analysis was to evaluate the difference in short-term recovery of lower-limb explosive performance between the control condition (Day 1) and the experimental NN condition (Day 2) during the 15-min post-exercise recovery window. Statistical significance was set a priori at an alpha level of α = 0.05.

The 95% Confidence Intervals were obtained as part of the descriptive statistics, calculated alongside the Mean ± Standard Deviation (SD) for the estimated effects (Jump Height Effect and Peak Power Effect). The 95% CIs were utilized to confirm the statistical significance of the intervention across the sample by verifying that the intervals for the metrics fell strictly above zero.

The parametric tests were applied to evaluate the Jump Height and Peak Power retention coefficients between the control and experimental conditions. Prior to applying these tests, the Shapiro–Wilk Test was employed initially to verify the assumption of normality for the distribution of paired differences: The first assumption is normality of differences. The second assumption is the absence of significant outliers in the differences. Third—continuous data level. Fourth—independence of observations.

A Paired Samples *t*-test was utilized to assess whether the mean difference between the paired Day 1 and Day 2 retention coefficients was statistically significantly different from zero. The paired *t*-test analysis comparing the Day 1 JH Coef*_N1_* and Day 2 JH Coef*_N2_* was calculated using Equation (7):

(7) $$t=\;\frac{\mathrm{XD}}{\mathrm{SD}/\sqrt{N}}$$

where *t* is the *t*-statistic, *XD* is the sample mean of the differences between the paired observations (e.g., Day 2 JH Coef*_N2_* minus Day 1 JH Coef*_N1_*), *SD* is the sample standard deviation of these differences, *N* is the number of pairs.

The *p*-value for a paired *t*-test was not calculated using a simple algebraic formula, but rather by finding the area under the curve of Student’s *t*-distribution. The *p*-value was determined using the cumulative distribution function of the *t*-distribution. If the *p*-value is less than 0.05, there is a statistically significant difference between the Day 1 and Day 2 Jump Height coefficients.

Cohen’s *d_z_* was calculated to determine the standardized mean difference and the magnitude of the intervention’s effect within the repeated-measures design. Effect Sizes Parametric (Cohen’s *d_z_*) of the effect for within-subject (paired) designs were calculated to determine the standardized mean difference and magnitude of the intervention using Equation (8):

(8) $$d_{z}\;=\;\frac{\mathrm{XD}}{\mathrm{SD}}$$

where *XD* represents the sample mean of the paired differences and *SD* represents the sample standard deviation of those differences.

Given the inherent constraints of a pilot study—specifically the small sample size of 8 athletes—non-parametric analyses were integrated to ensure statistical robustness and validate the parametric findings against potential outliers or subtle deviations from normality. We used the Wilcoxon signed-rank test as a conservative, non-parametric alternative to the paired *t*-test. This test ranks the absolute differences between pairs and does not assume a normal population distribution, making it highly reliable for small-*N* datasets. Wilcoxon Effect Size (*r*) is derived from the Z-score of the Wilcoxon test to provide a non-parametric measure of effect magnitude, ensuring the parametric effect sizes are not artificially inflated. The effect size is calculated as follows:

(9) $$r=\frac{\left|Z\right|}{\sqrt{N}}$$

In the context of paired non-parametric tests, the components of this equation are defined strictly by the data points that construct the underlying probability distribution. The sample consists of 8 athletes; each athlete undergoes assessment under two distinct conditions (Day 1 control and Day 2 experimental). Multiplying the participant count by the number of measurement instances yields *N* = 16 total observations.

## 3. Results

Comprehensive outcome data are summarized in Table 2.

Table 3 outlines the raw Jump Height (cm) and Peak Power (W/kg) measurements across both days, alongside the corresponding daily retention coefficients.

In Table 4 and Table 5, a positive value (Equations (5) and (6)) indicates that performance recovery differs between the control day and the subsequent NN day.

Prior to inferential analysis, the data were evaluated to ensure all assumptions for parametric testing were met. A Shapiro–Wilk test confirmed that the paired differences in Jump Height (JH) retention were normally distributed (*p* = 0.807). Inspection of the data confirmed an absence of extreme outliers, with the calculated JH differences clustering narrowly between −0.013 and 0.096. Furthermore, the dependent variables (lower-limb explosive performance retention coefficients) consist of continuous numerical data derived from objective, independently administered measurement protocols.

Descriptive statistics demonstrated a mean JH retention effect of 0.052 ± 0.034, with a 95% confidence interval (CI) strictly above zero [0.023, 0.081]. A paired-samples *t*-test comparing Day 1 and Day 2 retention coefficients revealed a statistically significant improvement in lower-limb explosive performance on the intervention day (*t* = 4.284, *p* = 0.0036). The standardized mean difference indicated a very large intervention effect (Cohen’s *d_z_* = 1.515). To ensure statistical robustness given the small sample size, a non-parametric Wilcoxon signed-rank test was also conducted; this analysis corroborated the primary parametric findings and confirmed a large non-parametric effect (Z = 2.38, *r* = 0.595).

Similarly, the intervention yielded a positive mean Peak Power (PP) retention effect of 0.032 ± 0.035 (95% CI [0.002, 0.062]). This result was also statistically significant (SE = 0.013, *t* = 2.545, *p* = 0.0384) and yielded a large effect size (Cohen’s *d_z_* = 0.900), further indicating a positive temporal association between the NN condition (Day 2) and the short-term recovery of lower-limb explosive performance.

## 4. Discussion

Initial statistical validation confirmed the dataset met requisite assumptions for parametric testing. Subsequent analyses revealed a statistically significant increase in Jump Height retention during the NN condition (Day 2) compared to the initial control condition (Day 1). This large effect was rigorously corroborated by supplementary non-parametric testing, ensuring robustness despite the small cohort. Parallel outcomes emerged in kinetic power metrics, where the NN condition temporally coincided with a statistically significant, large-magnitude enhancement in Peak Power retention. Collectively, these inferential findings support a positive temporal association between the NN protocol and the acute recovery of lower-limb explosive performance.

### 4.1. Natural Neurostimulation-Based Technique

This section synthesizes the theoretical rationale for the Natural Neurostimulation (NN) intervention. It contextualizes the NN technique within the broader literature, exploring how this biomimetic neurostimulation with specific parameters may facilitate neurophysiological processes that optimize efferent neuromuscular drive. Rooted in the evolutionary paradigm of maternal–fetal nervous system stimulation, the NN technique has been abstracted into a therapeutic approach [14] proposed to restore homeostasis within the clinical immuno-neuro-endocrine (CINE) axis. However, to maintain a strict demarcation between established theoretical frameworks and the current empirical findings, it is critical to emphasize that this study did not quantify direct neurophysiological or biochemical variables (e.g., EEG, cerebral oxygenation). Instead, the present empirical findings are explicitly limited to the acute preservation of lower-limb kinetic performance.

Drawing upon the broader literature to provide a mechanistic foundation, it is hypothesized that the NN intervention may facilitate the optimization of efferent motor drive, presenting a prospective countermeasure to central fatigue in athletic populations. Building upon this theoretical synthesis, Section 4.1 outlines the parameters of acoustic-photonic neurostimulation during task-induced brain activity that are hypothesized to elicit these beneficial effects. This rationale explores literature-based mechanisms across two scales: at the macroscopic level, it details the neurophysiological processes thought to drive temporal neuronal synchronization and cortical network activation; at the microscopic level, it explores the theoretical modulation of mitochondrial bioenergetics.

The NN technique uses weak, dual-modality acoustic-electromagnetic (specifically acoustic-photonic; photonic refers to the light spectrum of the EM field) stimulation, administered concurrently with task-induced brain activity through natural sensory processing. Rather than merely mimicking biological morphology, this NN approach relies on the systematic abstraction, mathematical modeling, and technological transfer of evolutionarily optimized NN mechanisms into a synthetic neurotherapeutic framework. Because the NN technique administers extremely weak transcranial electromagnetic radiation alongside bimodal sensory stimulation, comprising structured visual and acoustic inputs, it reduces the risk of excessive exposure to intense fields and ensures neurostimulation is localized to the neural networks associated with awareness. 

The heart’s EM field is a complex wave made up of a wide spectrum of extremely low frequencies from 0.003 Hz to 150 Hz, with a density of 10^−9^ T and 10^−12^ T at distances where EM waves cross the fetal brain. A massive spike in EM power is concentrated between 10 Hz and 50 Hz [11,12]. Maternal ventricular contraction and diaphragmatic excursions during breathing generate periodic mechanical pressure pulses in a range of 0.5–2 Hz. The heart delivers a complex acoustic wave that reflects the turbulence created when the heart valves snap shut. Normally, the fetus can perceive three of the mother’s heart tones during one phase of the heartbeat. Two normal heart sounds, *S*_1_ and *S*_2_, are produced by closing the atrioventricular and semilunar valves, respectively. The third heart sound (*S*_3_ protodiastolic gallop) is caused by the oscillation of blood back and forth between the walls of the ventricles initiated by blood rushing in from the atria. Phonocardiographic analysis at the myocardial surface demonstrated acoustic magnitudes exceeding 120 dB re 1 microPa for the first heart sound (*S*_1_), approximately 114 dB re 1 microPa for the second heart sound (*S*_2_), and approximately 110 dB re 1 microPa for the third heart sound (*S*_3_) [13]. 

To biomimetically emulate endogenous maternal cardiac activity, the NN protocol employs dual-modality acoustic-photonic stimulation delivered concurrently with task-induced cerebral activity. The visual component utilizes rapidly alternating photic stimulation across the visible spectrum, oscillating between short-wavelength (400 nm) and long-wavelength (700 nm) emissions. Concurrently, the acoustic modality delivers continuous auditory stimuli at a fundamental frequency of 1.3 Hz. The acoustic signature is a complex wave composed of three simultaneous sine waves: a primary carrier wave (*S*_1_ at 70 dB) overlaid with two supplementary harmonic profiles (*S*_2_ and *S*_3_, both at 21 dB). Operating within a subthreshold biophysical regime, the NN intervention is designed to theoretically emulate endogenous ephaptic coupling and sensory photic entrainment. Drawing upon established theoretical models rather than the current study’s biomechanical data, this mechanism is hypothesized to noninvasively promote temporal phase-locking. The subsequent sections delineate the empirical-based rationale for these selected stimulation parameters, outlining a biomimetic framework designed to replicate maternal neurostimulation.

#### 4.1.1. Cortical Phase-Locking via Photonic Inputs

The primary way pulsing light affects temporal coordination is through neural entrainment. When a rhythmic visual pulse hits the retina, it sends periodic streams of action potentials through the optic nerve to the primary visual cortex (V1) [24]. Rather than firing randomly, local cortical networks phase-lock their activity to the frequency of the incoming light pulses [25]. Unaveraged single-trial EEG analyses demonstrate that this macroscopic “entrainment” is actually composed of micro-level, transient bursts of synchronized neuronal activity occurring in precise alignment with the light transitions [26].

#### 4.1.2. Photoreceptor Activation

Alternating extreme wavelengths maximizes cortical power via distinct pathways [27,28]. Alternating between different wavelengths (e.g., swapping rapidly between blue and red light) introduces specific biophysical changes due to how our photoreceptors respond to color. The retina processes color via distinct cone pathways (S-cones for short-wavelength blue, M-cones for medium green, and L-cones for long-wavelength red). Alternating wavelengths force these separate pathways to switch back and forth from states of activation to sudden hyperpolarization: differential photoreceptor activation adaptations [27]. Recent literature shows that alternating between highly contrasting spectra—specifically combinations involving short-wavelength blue or long-wavelength red—generates significantly higher cortical power responses and entrainment amplitudes than pulsing mid-spectrum colors (like green and yellow). This occurs because short and long wavelengths drive higher Event-Related Potentials (ERPs) and activate broader frontal and middle cortical regions due to low mutual adaptation [28].

#### 4.1.3. Alternating Colors Neuromodulation Toward Cognitive Arousal

Color alternation decreases alpha inhibition and increases gamma synchronization [25]. The temporal coordination of alternating colors modifies specific brainwave frequencies to alter perception and states of consciousness. First, alternating pulses cause a power decrease in the lower alpha band (8–12 Hz). This drop in alpha power acts as a neural “gating mechanism”. It temporarily releases the cortex from inhibition, allowing incoming sensory data to pass through uninterrupted [25]. Concurrently, there is a sharp increase in gamma-band frequencies (>30 Hz). Gamma oscillations are responsible for binding different features of a visual object (like shape and color) together. The alternating wavelengths force local gamma networks to rapidly synchronize to construct a coherent perceptual stream of the changing colors [25].

Beyond the visual cortex, pulsating color wavelengths modulate overall brain state through non-visual, subcortical pathways. Research indicates that flickering light stimulation (specifically at therapeutic frequencies like 40 Hz) triggers a rapid and sustained increase in extracellular adenosine levels in the visual cortex. This process is highly dependent on light frequency and wavelength, modulating primary visual cortical activity to promote states like sleep onset or heightened focus [29].

Short-wavelength pulsing (blue light) directly activates intrinsically photosensitive retinal ganglion cells (ipRGCs). This pathway projects to the suprachiasmatic nucleus (SCN), suppressing melatonin secretion and altering the neural activity of stress and arousal regions like the paraventricular nucleus (PVN). This shifts global network excitability, speeding up attention function and cognitive reaction times [30,31].

#### 4.1.4. Bioenergetic Optimization via Acoustic Mechanotransduction

Cardiovascular-mimetic acoustic waveforms generate cyclic pressure oscillations that alter plasma membrane tension at the cellular level, inducing the gating of mechanosensitive ion channels [32,33]. Specifically, mechanical deformation of the lipid bilayer activates Piezo2 channels, driving a regulated influx of extracellular Ca^2+^ and Na^+^ cations [32,33]. This mechanotransductive cascade transduces macroscopic hydrodynamic displacement into localized receptor potentials and subsequent membrane depolarization [32,33].

Importantly, this mechanotransductive signaling axis directly modulates intracellular bioenergetics. Biophysical studies indicate that physiological low-frequency mechanical stimulation (~1 Hz)—corresponding to maternal hemodynamic and cardiac frequencies—regulates mitochondrial respiratory chain dynamics and cellular energetic homeostasis [34]. Neural populations sense these ~1 Hz cyclic mechanical perturbations through mechanosensitive elements, in which transient intracellular calcium elevation acts as an upstream regulator of mitochondrial function.

By elevating mitochondrial adenosine triphosphate (ATP) synthesis and maintaining intracellular calcium homeostasis, these low-frequency mechanical oscillations sustain activity-dependent neurotransmitter exocytosis. This mechanically driven energetic optimization modulates synaptic plasticity mechanisms, thereby supporting structural neural circuit refinement and cognitive performance.

#### 4.1.5. Acoustic and Photonic Bioenergetic Optimization for Beneficial Neuroplasticity

Literature elucidates that both sensory modalities of this bicomponent NN intervention synergistically alter mitochondrial function to drive neural adaptation [34,35,36]. First, wavelength-specific photic stimulation modulates neuronal oscillatory activity across diverse cortical networks by directly upregulating mitochondrial adenosine triphosphate (ATP) production within the retina’s image-forming and ganglion cell pathways [35]. Second, the acoustic modality provides a parallel stimulus that directly modulates mitochondrial bioenergetics—optimizing non-convective energy transfer, ATP synthesis, calcium buffering, and ROS signaling requisite for synaptic neurotransmission [34,36]. This sound-induced alteration in mitochondrial activity serves as a primary neurostimulatory mechanism, regulating the synthesis and release of key neurotransmitters such as dopamine, serotonin, and corticosterone. Furthermore, low-frequency acoustic oscillations leverage these bioenergetic shifts to positively modulate hippocampal synaptic plasticity, thereby enhancing memory and broader cognitive functions [37].

Mitochondria are highly proteome-efficient in generating ATP, providing energy for normal functioning [38]. Mitochondrial ATP generation is even more proteome-efficient than glycolysis [16]. These organelles produce energy in oxidative phosphorylation: the electron transport chain and the enzyme ATP synthase convert oxygen and nutrients into ATP. Research shows that mitochondria synthesize ATP when mixed with a light-capturing metabolite of chlorophyll [39]. Moreover, research shows that red light (650–800 nm) affects mitochondrial complex IV (cytochrome oxidase), thereby increasing ATP release [40]. Large numbers of mitochondria exist in the inner cells of the retina. At the same time, mitochondrial output signals regulate the functions of other organelles and systemically tune physiology by integrating information through dynamic, network-based physical interactions and diffusion mechanisms [41]. According to Picard and Shirihai [41], mitochondria constitute the mitochondrial information processing system (MIPS), providing signal processing and integration [41]. Mitochondria, commonly found in synaptic terminals, play a crucial role in maintaining neurotransmission by producing ATP and buffering Ca^2+^. This underscores their importance in neurological function [42].

#### 4.1.6. NN Intervention for Oxygenation

Moreover, because the acoustic and visual components of NN stimulate mitochondrial activity, this facilitates brain tissue oxygenation, as increased mitochondrial ATP production within endothelial cells (the inner lining of blood vessels) is required to relax and dilate the vessels [43,44]. Vascular research has shown that healthy mitochondrial ATP production in the endothelium actively drives vasodilation [45]. When endothelial mitochondria produce sufficient ATP, the cells can successfully activate Endothelial Nitric Oxide Synthase. This causes a surge in Nitric Oxide release, which relaxes the surrounding smooth muscle and dilates the vessel [46,47]; this process is known as endothelium-dependent vasodilation [48]. Therefore, the acoustic and visual components of NN provide brain tissue oxygenation, facilitating neuroplasticity.

#### 4.1.7. Experience-Dependent Cortical Plasticity

Evidence shows that engaging in visual-acoustic stimulation based on cognitive computer games can lead to significant structural increases in the brain, sometimes occurring rapidly [49]. Research indicates that measurable neuronal structural changes due to neuroplasticity can appear within 2–3 months of such systematic impacts. A review of the scientific literature has shown that the systematic effect of cognitive load from computer work, with a total training duration of 16 h (a few hours of weekly exercise), can affect brain structure and function, depending on how the impact is delivered [50]. Research has documented an increase in grey matter thickness following a systematic three-month training course comprising 26 h of exercises [51]. These experimental data show that the cognitive load generated by computer devices by means of a monitor with photonic radiation in the diapason 380–780 nm, the total irradiance falling on a surface at a 50 cm distance ranges between 0.5 and 2.0 W/m^2^ (or 50 to 200 µW/cm^2^), and the electromagnetic density of 10^−2^ T can affect brain structure and function when used systematically for more than 26 h [51]. Although these studies observed neurostimulation-induced alterations in brain structure during multiple sessions of task-related mental activity, they did not systematically evaluate specific stimulation parameters or their associated clinical efficacy. Consequently, while these data confirm macroscopic structural plasticity, they preclude a definitive analysis of the dosimetric parameters required to achieve targeted therapeutic outcomes. 

Synthesizing the physiological mechanisms delineated in Section 2.3, acoustic-photonic neurostimulation is hypothesized to operate at both cellular and systemic levels. At the micro-level, the NN intervention is proposed to modulate mitochondrial bioenergetics by optimizing non-convective energy transfer, ATP synthesis, calcium buffering, and reactive oxygen species (ROS) signaling—processes essential for sustained synaptic neurotransmission. At the macro-level, NN technique administered concurrently with task-induced cognitive activity is hypothesized to drive temporal neuronal synchronization, activate cognitive cortical networks, and restore overall neural homeostasis. Furthermore, NN intervention is theorized to enhance cerebral oxygenation via endothelium-dependent vasodilation, upregulate synaptic protein synthesis [14], and normalize the release of inhibitory neurotransmitters and hormones. In the context of neuromuscular recovery, these neuroendocrine shifts are proposed to directly augment efferent signaling along the neuromuscular axis. Consequently, these hypothesized pathways for central-to-peripheral neural integration provide a theoretical basis for the acute preservation of lower-limb kinetic performance observed in the current study, offering a prospective mechanism for mitigating exercise-induced central fatigue.

Preliminary empirical evidence suggests that longitudinal application of NN neurotherapy—typically administered as a 20-min daily intervention over several months—may be associated with positive clinical outcomes across various conditions, including migraines [52], cerebral microadenomas, neurodegenerative disorders [53], and dysmenorrhea [54,55].

### 4.2. Single-Arm Fixed-Sequence Design

We selected a fixed-condition sequence as a methodologically suitable approach for this pilot study, given the technical challenges of isolating the current and residual effects of the NN condition on participants. Specifically, the present case report was designed to offer descriptive insights rather than establish causal inference. Case studies in sports science serve primarily as proof-of-concept, feasibility, or diagnostic observations rather than definitive causal trials. While a fixed control->experimental design cannot statistically rule out order or habituation effects, it provides high ecological and descriptive value necessary to justify larger, randomized crossover RCTs [56,57,58]. Then, in small sample size designs, each participant acts as their own control. The core objective of single-case and small-series methodology is tracking individual biological/behavioral shifts across stable baseline and intervention phases. Because the analytical focus is idiographic (individual patterns over time) rather than nomothetic (group averages), establishing a reliable, unperturbed baseline is methodologically sufficient to observe marked intra-individual deviations when the active condition is introduced [58,59]. Finally, this design minimizes the risk of residual neurostimulation carryover effects; a fixed sequence is required when the intervention entails non-withdrawable physiological changes. If the NN condition were performed before the control condition, it could alter corticospinal excitability and metabolic depletion, which may persist for days and potentially affect the control-condition data.

We deliberately adopted an open-label design to minimize the residual carryover effects of the NN intervention—among other reasons detailed below—opting for a protocol without a sham-controlled condition. The current pilot, small-cohort study (8 participants) was primarily designed to establish preliminary safety, operational viability, protocol tolerance, and early signals of utility rather than definitive population-level proof of efficacy. Implementing a sham condition requires additional experimental arms, double-blind setups, and extended testing sessions, which unnecessarily increases participant burden and logistical complexity during initial exploratory stages [58,59]. Moreover, statistically evaluating placebo effects requires large, well-powered sample sizes to detect subtle between-condition variances. In case studies (e.g., with 8 participants), statistical power is inherently low; attempting to isolate true intervention effects from sham/placebo effects mathematically yields imprecise confidence intervals and unreliable effect size estimates. Therefore, small studies prioritize direct observation of large physiological signals relative to baseline rather than attempting underpowered sham comparisons [59]. Additionally, for NN intervention, creating a convincing, sensory-identical yet biologically inert sham is methodologically challenging [58,60].

The fixed-condition sequence and the absence of a sham condition constrain the interpretation of this pilot study’s results.

### 4.3. Justification of Measurement Intervals and Exclusion of T2

We performed three jump assessments each experimental day: baseline (T1), immediately after the exercise round (T2), and after the 15-min recovery period (T3). The measurement (T2) served solely as an immediate physiological manipulation check to confirm that the upper-body loading protocol was of sufficient intensity to elicit a systemic neuromuscular response. However, T2 data were excluded from the final lower-limb explosive performance analysis because immediate post-exercise measurements are confounded by acute physiological responses—namely, PAPE and catecholamine surges. Measurements obtained immediately following physical exertion do not accurately assess neuromuscular fatigue [21,22]. First, immediately after, and for up to 7 min after, high-intensity loading, the neuromuscular system experiences a transient enhancement in function known as Post-Activation Potentiation (PAP) or PAPE. In the VALD jump test, PAPE acts as a confounding variable. It creates a “masking effect” in which acute potentiation temporarily hides central and peripheral nervous system fatigue. High-intensity exercise increases the phosphorylation of myosin regulatory light chains within the muscle, making them highly sensitive to calcium. The exercise acutely elevates muscle temperature and increases motor neuron excitability. Because of these acute physiological boosts, explosive performance can actually increase immediately post-workout [53]. Second, intense exercise strongly activates the sympathetic nervous system, acting as a major physiological stressor that stimulates catecholamine surges (such as Adrenaline, Dopamine, and Noradrenaline) that can be 1.5 to over 20 times higher than resting baseline levels [23,61]. Third, the study aimed to evaluate athletes’ performance using the decoupled jump test methodology to determine their recovery. Therefore, the study was designed to provide a comparative analysis of pre- and post-exercise data, using bilateral force-plate measurements from before (T1) and 15 min after the workout (T3), rather than between T2 and T3.

### 4.4. Assessing Central Fatigue in a Decoupled Bilateral Force-Plate Protocol

Research evidence demonstrates that vertical jump assessments in Countermovement Jump design administered on VALD ForceDecks dual force-plate systems offer high concurrent validity and test–retest reliability for evaluating neuromuscular fatigue [6,7]. The literature highlights that beyond macro-level output metrics like jump height, automated force-time curve decomposition allows researchers to detect residual neuromuscular fatigue and altered movement strategies following high-intensity external loading protocols [8,9].

This study used decoupled bilateral force-plate methodology; it employed upper-body loading with concurrent lower-limb kinetic assessment to distinguish central from peripheral fatigue. As the lower limbs were not subjected to direct metabolic depletion, reductions in jump height and peak power at T3 on the control day can be attributed to systemic central motor unit recruitment deficits. A review of 35 studies and 58 outcome measures demonstrates that fatiguing exercise in one muscle group (e.g., upper limbs) decreases voluntary activation and motor unit recruitment in remote, non-exercised muscle groups (e.g., lower limbs), predominantly via central nervous system inhibition rather than peripheral metabolic factors [10]. This phenomenon, termed non-local muscle fatigue (NLMF), manifests as impaired performance in contralateral or distant muscles following localized fatigue protocols. Therefore, the observed attenuation in lower-limb kinetic output at T3 is interpreted as a potential proxy for central fatigue.

### 4.5. Limitations

The study used a fixed experimental order, which limited the results interpretation because the better result on Day 2 might partly reflect familiarization with the VALD ForceDesck testing procedure, learning effects, or differences in recovery status rather than the NN intervention. Although the cohorts demonstrated clinical equivalence at baseline, the non-randomized allocation introduces potential unobserved confounding variables—such as baseline motivation—that cannot be entirely mitigated. 

The absence of a sham/placebo condition is another limitation. The NN intervention was highly noticeable, so participants presumably knew when they were receiving it. Without a sham condition, expectancy, arousal, motivation, and placebo-related effects cannot be excluded as contributors to the subsequent jump performance. It is also impossible to determine whether any observed effect resulted from the proposed NN mechanism, from sensory stimulation, cognitive engagement, expectancy/placebo effects, or simply differences between active and passive recovery. In large, randomized crossover RCTs, these arguments substantially limit causal interpretation, meaning that the intervention itself was not definitively responsible for the observed improvement. 

### 4.6. Future Research

Future investigations must employ randomized, sham-controlled designs to validate these preliminary findings and establish definitive causal inference. To enhance external validity, subsequent research should include female cohorts. Furthermore, longitudinal studies extending beyond a three-month follow-up are necessary to evaluate the efficacy of the NN intervention in mitigating cumulative central fatigue across successive athletic competitions. Finally, integrating electroencephalographic (EEG) assessments with sensorimotor data will be critical to determine whether functional performance enhancements directly correlate with underlying neurophysiological and structural adaptations.

## 5. Conclusions

In this pilot study, the Natural Neurostimulation (NN) intervention was associated with statistically significant, large-magnitude improvements in both Jump Height and Peak Power retention coefficients following fatiguing exercise. While these findings suggest that the NN protocol may facilitate the short-term recovery of lower-limb explosive performance, the absence of randomization and a sham control precludes establishing a definitive causal relationship. Nevertheless, these preliminary results demonstrate the operational feasibility of the intervention, providing a clear methodological foundation for future fully powered, randomized controlled trials.

## Figures and Tables

**Table 1:** The Load Training Protocol: Exercise Round Lasts 35 min.

Step	Exercise	Load % 1RM	Target Volume	Rest Interval
Ex. 1	Dumbbell	75% 1RM	4 sets × 10–12 reps (to technical failure)	90 s
Ex. 2	Lat Pulldown	75% 1RM	4 sets × 10–12 reps (Reps in Reserve: 0)	90 s
Ex. 3	Seated Dumbbell Overhead Press	70% 1RM	3 sets × 12 reps	60 s
Ex. 4	Seated Cable Row	70% 1RM	3 sets × 12 reps	60 s
Ex. 5	Finisher: Push-ups	Bodyweight	3 sets × Maximum reps possible	45 s

**Table 2:** T1, T2, and T3 Results (Day 1 & Day 2).

Code	Day	Measurement	Jump Height	Peak Power (W/kg)
P(1)	1	T1	36.0	54.9
P(1)	1	T2	34.1	53.3
P(1)	1	T3	34.0	52.0
P(1)	2	T1	33.7	52.4
P(1)	2	T2	34.6	52.6
P(1)	2	T3	34.2	52.2
P(3)	1	T1	37.6	54.1
P(3)	1	T2	36.1	53.0
P(3)	1	T3	31.0	49.0
P(3)	2	T1	37.8	50.8
P(3)	2	T2	35.3	51.7
P(3)	2	T3	34.8	49.2
P(4)	1	T1	35.5	52.5
P(4)	1	T2	36.6	52.0
P(4)	1	T3	34.3	49.7
P(4)	2	T1	34.2	48.5
P(4)	2	T2	37.0	52.9
P(4)	2	T3	32.6	46.1
P(5)	1	T1	45.8	60.0
P(5)	1	T2	46.3	63.1
P(5)	1	T3	45.2	59.9
P(5)	2	T1	40.8	57.1
P(5)	2	T2	43.9	60.7
P(5)	2	T3	42.1	60.9
P(6)	1	T1	34.5	50.7
P(6)	1	T2	34.4	50.6
P(6)	1	T3	32.4	50.2
P(6)	2	T1	33.8	48.5
P(6)	2	T2	36.7	52.2
P(6)	2	T3	32.8	47.3
P(7)	1	T1	31.5	51.8
P(7)	1	T2	34.6	53.5
P(7)	1	T3	30.1	51.6
P(7)	2	T1	30.2	49.5
P(7)	2	T2	35.0	55.7
P(7)	2	T3	30.8	50.6
P(8)	1	T1	33.7	53.5
P(8)	1	T2	32.8	53.0
P(8)	1	T3	31.7	50.8
P(8)	2	T1	31.0	50.6
P(8)	2	T2	31.8	51.9
P(8)	2	T3	31.7	51.6
P(10)	1	T1	34.5	52.3
P(10)	1	T2	34.5	53.6
P(10)	1	T3	32.7	51.8
P(10)	2	T1	33.1	51.2
P(10)	2	T2	33.6	52.0
P(10)	2	T3	32.7	50.2

The abbreviation P (*N*) means the results of the *N* athlete; T1, T2, and T3 are the VALD measurements (T1 before the exercise, T2 immediately after the exercise round, and T3 after the 15-min recovery period).

**Table 3:** Comprehensive Results & Retention Coefficients (Day 1 & Day 2).

Code/Day	Jump Height (cm)	JH Coef	Peak Power (W/kg)	PP Coef
P(1)_1_^1^ baseline	36.0	1.000	54.9	1.000
P(1)_1_^2^	34.0	0.944	52.0	0.947
P(1)_2_^1^ baseline	33.7	1.000	52.4	1.000
P(1)_2_^2^	34.2	1.015	52.2	0.966
P(3)_1_^1^ baseline	37.6	1.000	54.1	1.000
P(3)_1_^2^	31.0	0.824	49.0	0.906
P(3)_2_^1^ baseline	37.8	1.000	50.8	1.000
P(3)_2_^2^	34.8	0.921	49.2	0.969
P(4)_1_^1^ baseline	35.5	1.000	52.5	1.000
P(4)_1_^2^	34.3	0.966	49.7	0.947
P(4)_2_^1^ baseline	34.2	1.000	48.5	1.000
P(4)_2_^2^	32.6	0.953	46.1	0.951
P(5)_1_^1^ baseline	45.8	1.000	60.0	1.000
P(5)_1_^2^	45.2	0.987	59.9	0.998
P(5)_2_^1^ baseline	40.8	1.000	57.1	1.000
P(5)_2_^2^	42.1	1.032	60.9	1.067
P(6)_1_^1^ baseline	34.5	1.000	50.7	1.000
P(6)_1_^2^	32.4	0.939	50.2	0.990
P(6)_2_^1^ baseline	33.8	1.000	48.5	1.000
P(6)_2_^2^	32.8	0.970	47.3	0.975
P(7)_1_^1^ baseline	31.5	1.000	51.8	1.000
P(7)_1_^2^	30.1	0.956	51.6	0.996
P(7)_2_^1^ baseline	30.2	1.000	49.5	1.000
P(7)_2_^2^	30.8	1.020	50.6	1.022
P(8)_1_^1^ baseline	33.7	1.000	53.5	1.000
P(8)_1_^2^	31.7	0.941	50.8	0.950
P(8)_2_^1^ baseline	31.0	1.000	50.6	1.000
P(8)_2_^2^	31.7	1.023	51.6	1.020
P(10)_1_^1^ baseline	34.5	1.000	52.3	1.000
P(10)_1_^2^	32.7	0.948	51.8	0.990
P(10)_2_^1^ baseline	33.1	1.000	51.2	1.000
P(10)_2_^2^	32.7	0.988	50.2	0.980

The abbreviation P (*N*)*_x_^y^* means the results of the *N* athlete on the *y* VALD measurement (^1^ 1st [before the exercise] or ^2^ 2nd [15 min after the exercise]) on the *x* day (1st or 2nd).

**Table 4:** Jump Height & Jump Height Effect.

Code	Day 1 JH Coef*_N1_*	Day 2 JH Coef*_N2_*	JH Effect (*N*) (Day 2—Day 1)
P(1)	0.944	1.015	+0.070
P(3)	0.824	0.921	+0.096
P(4)	0.966	0.953	−0.013
P(5)	0.987	1.032	+0.045
P(6)	0.939	0.970	+0.031
P(7)	0.956	1.020	+0.064
P(8)	0.941	1.023	+0.082
P(10)	0.948	0.988	+0.040

**Table 5:** Peak Power & Fatigue Effect.

Code	Day 1 PP Coef*_N1_*	Day 2 PP Coef*_N2_*	PP Effect (*N*) (Day 2—Day 1)
P(1)	0.947	0.996	+0.049
P(3)	0.906	0.969	+0.063
P(4)	0.947	0.951	+0.004
P(5)	0.998	1.067	+0.068
P(6)	0.990	0.975	−0.015
P(7)	0.996	1.022	+0.026
P(8)	0.950	1.020	+0.070
P(10)	0.990	0.980	−0.010

## Data Availability

The original contributions presented in this study are included in the article. Further inquiries can be directed to the corresponding author.
